# Correlation of Endocrine Disrupting Chemicals Serum Levels and White Blood Cells Gene Expression of Nuclear Receptors in a Population of Infertile Women

**DOI:** 10.1155/2013/510703

**Published:** 2013-04-21

**Authors:** Donatella Caserta, Francesca Ciardo, Giulia Bordi, Cristiana Guerranti, Emiliano Fanello, Guido Perra, Francesca Borghini, Cinzia La Rocca, Sabrina Tait, Bruno Bergamasco, Laura Stecca, Roberto Marci, Giuseppe Lo Monte, Ilaria Soave, Silvano Focardi, Alberto Mantovani, Massimo Moscarini

**Affiliations:** ^1^Department of Obstetrics and Gynaecological Sciences and Urological Sciences, University of Rome “Sapienza”, S. Andrea Hospital, Via di Grottarossa 1035, 00189 Rome, Italy; ^2^Department of Woman Health and Territory's Medicine, University of Rome “Sapienza”, S. Andrea Hospital, Via di Grottarossa 1035, 00189 Rome, Italy; ^3^Department of Environmental Sciences “G. Sarfatti”, University of Siena, Via P.A. Mattioli 4, 53100 Siena, Italy; ^4^Department of Food Safety and Veterinary Public Health, Food and Veterinary Toxicology Unit, Istituto Superiore di Sanità, Viale Regina Elena 299, 00161 Rome, Italy; ^5^Department of Biomedical Sciences and Advanced Therapies, Section of Obstetrics and Gynaecology, University of Ferrara, Corso Giovecca 203, 44121 Ferrara, Italy

## Abstract

Significant evidence supports that many endocrine disrupting chemicals could affect female reproductive health. Aim of this study was to compare the internal exposure to bisphenol A (BPA), perfluorooctane sulphonate (PFOS), perfluorooctanoic acid (PFOA), monoethylhexyl phthalate (MEHP), and di(2-ethylhexyl) phthalate (DEHP) in serum samples of 111 infertile women and 44 fertile women. Levels of gene expression of nuclear receptors (ER**α**, ER**β**, AR, AhR, PXR, and PPAR**γ**) were also analyzed as biomarkers of effective dose. The percentage of women with BPA concentrations above the limit of detection was significantly higher in infertile women than in controls. No statistically significant difference was found with regard to PFOS, PFOA, MEHP and DEHP. Infertile patients showed gene expression levels of ER**α**, ER**β**, AR, and PXR significantly higher than controls. In infertile women, a positive association was found between BPA and MEHP levels and ER**α**, ER**β**, AR, AhR, and PXR expression. PFOS concentration positively correlated with AR and PXR expression. PFOA levels negatively correlated with AhR expression. No correlation was found between DEHP levels and all evaluated nuclear receptors. This study underlines the need to provide special attention to substances that are still widely present in the environment and to integrate exposure measurements with relevant indicators of biological effects.

## 1. Introduction

An endocrine disrupting compound (EDC) is defined as “an exogenous agent that interferes with synthesis, secretion, transport, metabolism, binding action, or elimination of natural blood-borne hormones that are present in the body and are responsible for homeostasis, reproduction, and developmental process” [1].

The homeostasis of sex steroids and the thyroid are the main targets of EDC effects; hence, reproductive health, considered as a continuum from gamete production and fertilization through to intrauterine and postnatal development of progeny, is recognized as being especially vulnerable to endocrine disruption [2].

This study stems from the PREVIENI project (http://www.iss.it/prvn/), founded by the Italian Environment Ministry. Aim of the project is to integrate biomarkers of chemical exposure, biologically effective dose biomarkers, and clinical findings, in order to assess the relationship between reproductive health and emerging EDC that are still incompletely considered in the environment and health surveillance.

This study presents the data on internal EDC exposure levels in infertile women from three different IVF units as well as the expression of nuclear receptors (NRs) in peripheral blood mononuclear cells (PBMCs), as biomarkers of effective dose.

Molecular targets were bisphenol A (BPA), perfluorooctane sulphonate (PFOS), perfluorooctanoic acid (PFOA), monoethylhexyl phthalate (MEHP), and di(2-ethylhexyl) phthalate (DEHP).

Bisphenol A (BPA) is primarily used in the manufacture of polycarbonate plastic sheets and epoxy resins present in various consumer products, such as food contact materials [3]. BPA migration from the plastic polymers leads to contamination of both food and environment.

Perfluooctane sulphonate (PFOS) and perfluorooctanoic acid (PFOA) are widely present in consumer's products, including textiles, films, and electric materials. Dietary exposure may be also considerable, in particular through food of animal origin, such as fish [4].

Di(2-ethylhexyl)-phthalate (DEHP) is a major representative of phthalates, widely used plasticizer used also in cosmetics, personal care products, and food packaging materials [5]. Mono(2-ethylhexyl)-phthalate (MEHP) is the major toxicologically relevant metabolite of DEHP [6]. Human exposure to phthalates may occur through diet or indoor environment.

The panel of NRs includes estrogen receptor alpha (ER*α*) and beta (Er*β*), androgen receptor (AR), pregnane X receptor (PXR), aryl hydrocarbon receptor (AhR), and peroxisome proliferator-activated receptor gamma (PPAR*γ*).

## 2. Materials and Methods

From January 2009 to April 2010, 111 women aged 18–40 years and affected by primary infertility were enrolled in the study. They were examined and admitted to three IVF units:

 
*n* = 50: Department of Women Health and Territory's Medicine of “Sapienza,” “S. Andrea” Hospital, University of Rome.
 
*n* = 38: Department of Biomedical Sciences and Advanced Therapies, Section of Obstetrics and Gynaecology, University of Ferrara.
 
*n* = 33: Infertility Center S.T.S. of Sora.

Forty-four fertile women aged 18–40 years with regular menstrual cycle who obtained a spontaneous pregnancy in the last year and stopped breastfeeding at least six months before were enrolled as control group. Women were asked to fill out a questionnaire about their life habits (alcohol, smoke, and diet), age, parity, work, residence, and previous diseases. Patients reporting smoking habit, vegetarian diet, occupational exposure to EDC, body mass index (BMI) > 30, inflammatory or infectious diseases, and diagnosis of male infertility factor were excluded from the study.

This work has been carried out in accordance with the Code of Ethics of the World Medical Association (Declaration of Helsinki) for experiments involving humans. Approval from the ethical committee of Department of Woman Health and Territory's Medicine, University of Rome “Sapienza” had been obtained before the beginning of this study, and all patients subscribed an informed consensus.

### 2.1. Collection and Storage of Samples

A 20 mL sample of venous blood was collected from each woman. All samples obtained from infertile women were collected before hormonal stimulation. Each blood sample was divided into three parts: two 5 mL aliquots of heparin-treated whole blood and a 10 mL aliquot centrifuged to obtain serum. Serum and a 5 mL aliquot of heparin-treated whole blood were then frozen and sent to the Environmental Sciences Department “G. Sarfatti” of University of Siena for the chemical analyses to detect EDC levels. Heparin-treated whole blood from IVF Rome and whole blood collected by PAXgene tubes from IVF Ferrara and Sora were sent to the Department of Veterinary Public Health and Food Safety, Istituto Superiore di Sanità in Rome to assess NR expression levels.

### 2.2. Chemical Analyses

BPA, PFOS, PFOA, DEHP, and MEHP were extracted using a liquid-liquid separation procedure and measured through a high-performance liquid chromatography (HPLC) with electrospray ionization (ESI) tandem mass spectrometry. For each substance, the limit of detection (LOD) corresponded to the respective measurement value in blanks + 3DS.

The extraction procedure of BPA follows the procedure previously described by Prins et al. [7]. Samples were defrosted, and 2 *μ*L/mL of the hydrolytic enzyme glucuronidase were added to each 0.5 mL of serum. The sample was mixed and incubated at 37°C for 12 hours. Three mL of diethyl ether were then added, and the sample was mixed up for 30 minutes and centrifuged for 5 minutes at 4000 rpm. The fluid part was collected in a 15 mL BPA-free vial. This procedure was repeated three times. Solvent evaporated at room temperature with a light nitrogen flow, and the deposit was reconstituted with 0.5 mL of acetonitrile and then filtered through a nylon filter with 0.2 *μ*m pores; 0.5 mL of acetonitrile were added, and the sample was then collected in autosampling vials. Analytical separation was performed according to Coughlin et al. [8]: 20 *μ*L were injected, by autosampling, in the HPLC column (C18 Betasil C18, 50 mm × 2.1 mm Ø, 5 *μ*m of inside thickness film) with a 250 *μ*L/minutes flow. The LOD for BPA was 0.5 ng/mL.

The analytical procedure for PFOS and PFOA follows Governini et al. [9]. The blood sample was defrosted, and then 0.5 mL of serum or whole blood, 1 mL of tetrabutylammonium hydrogen sulfonate 0.5 MpH 10, and 2 mL of sodium carbonate buffer 0.25 M were added in a 15 mL polypropylene tube. After mixing, 5 mL of methyl-tert-butyl ether (MTBE) were added and mixed again for 20 minutes. The sample was centrifuged at 3500 rpm for 25 minutes in order to separate the organic part (ether) from the fluid part. A volume of 4 mL of the organic part was collected in a 15 mL tube. The fluid part was treated with 4 mL MTBE, mixed up for 20 minutes, and centrifuged for 25 minutes at 3500 rpm in order to separate organic part by the remaining fluid part. Solvent evaporated at room temperature with a light nitrogen flow, and the deposit was reconstituted with 0.5 mL of methanol. The sample was then mixed by vortexing for 30 seconds, filtered through a nylon filter pores of 0.22 *μ*m diameter, and collected in autosampling vials. Analytical separation was performed by Finnigan LTQ Thermo Electron Corporation. Twenty *μ*L were injected, by autosampling, in the HPLC column (C18 Betasil C18, 50 mm × 2.1 mm Ø, 5 *μ*m of inside thickness film); column temperature was kept at constant temperature (30°C), and the substances of interest were separated by a mobile phase of ammonium/acetate/methanol 2 mM with a 300 *μ*L/minutes flow. After 10 minutes chromatographic running, HPLC was interfaced, through an electrospray ionization (ESI) source, working in negative mode, to a mass spectrometer at triple linear quadrupoles. Ions used for identifying PFOS and PFOA were 412,8 > 168.8, 218.8, and 498.8 > 368.9, respectively. For the quantitative analysis, a four-point calibration curve, obtained by progressive methanol dilution of a standard solution with relevant analytes (Chiron, Trondheim, Norway), was used. The LOD for PFOS and PFOA was 0.4 ng/mL.

The extraction procedure of DEHP and MEHP follows the protocol described by Takatori et al. [10]. After thawing, 4 mL of acetone were added to 0.5 mL of serum, mixed up for 5 minutes, and centrifuged at 3000 rpm for 20 minutes. The fluid part was collected in a 15 mL tube, treated with 1 mL of acetone, mixed up for 5 minutes, and centrifuged for 20 minutes at 3000 rpm in order to complete the separation of the organic part from the fluid part. Solvent evaporated at room temperature with a light incomplete nitrogen flow, and the deposit was reconstituted with 0.5 mL of acetonitrile. The sample was filtered through a nylon filter pores of 0.22 *μ*m diameter and collected in autosampling vials. Twenty *μ*L were injected in the HPLC and analyzed using an ODS-2 Hypersil 150 × 2.1 mm column with a flow of 200 *μ*L/mL. The LOD was 2 ng/g for MEHP and 10 ng/g for DEHP.

Glass vials were used in order to avoid possible release of EDC from plastic materials. In addition, to avoid contamination, all materials were rinsed with methanol. Data quality assurance and quality control protocols included matrix spikes, analyses of laboratory blanks, and continuous verification of the calibration. Blanks were analyzed with a set of six samples as a check for possible laboratory contamination and interferences.

### 2.3. Expression of Nuclear Receptors

NR gene expression was assessed on PBMC. PBMCs were separated from whole blood by Ficoll-Hypaque density centrifugation from heparin-treated samples from IVF Rome and extracted for their total RNA content by the RNeasy Mini Kit (QIAGEN). Samples from IVF Ferrara and Sora were extracted for their total RNA content by the PAXgene Blood RNA Kit (PreAnalytiX GmbH, 8634 Hombrechtikon, CH). RNA samples were quantified by NanoDrop. One *μ*g of total RNA from each sample was retrotranscribed to cDNA by the cDNA Synthesis Kit (Quantace). Gene expression analysis was performed by quantitative real-time PCR using the SensiMix SYBR Kit (Quantace). GAPDH was used as reference gene. Specific primers for the selected NR and GAPDH were designed using the Primer-BLAST web application and purchased by Life Technologies. Real-time PCR reactions were run on a Stratagene MP3005P Thermocycler. Gene expression levels were reported as 2exp(ΔCt).

### 2.4. Statistical Analysis

Analysis of data was performed using the Statistical Package for Social Sciences (SPSS) version 16.0 for Windows (SPSS, Chicago, USA). Normally distributed data were analyzed by the Student's *t*-test. The Mann-Whitney *U* test for continuous non-parametric variables was used to evaluate group differences in NR. *χ* ^2^ and Fisher test were used for comparison of rates and proportions. Pearson's test was used to demonstrate correlations between EDC levels and nuclear receptors expression and between age and BMI with EDC and nuclear receptors expression. *P* values <0.05 were defined as statistically significant.

## 3. Results

The mean age of infertile women group was 35.3 ± 0.4 years and was comparable to the mean age of the control group (34.8 ± 4.6 years). Mean BMI of the infertile group did not significantly differ from the control group (23.44 ± 0.4 and 23.24 ± 0.7 kg/m^2^, resp.). The following infertility factors were diagnosed in the study group: infertility sine causa (*n* = 34, 31.5%), tubal infertility (*n* = 23, 20.7%), immunological infertility (*n* = 17, 15.3%), thyroid dysfunction (*n* = 15, 13.5%), endometriosis (*n* = 11, 10%), polycystic ovarian syndrome (*n* = 7, 6.4%), and reduced ovarian reserve (*n* = 4, 3.6%). In the control group, 26 women had their first pregnancy in the last year, whereas 12 had their second pregnancy and 6 their third pregnancy before enrollment.

Table 1 shows the distribution of frequency of blood samples with EDC levels above LOD in both infertile and fertile groups. The percentage of women with BPA serum levels above the LOD was significantly higher in the infertile group (50/111, 45%, range 0.5–133.5 ng/mL) than in the control group (10/44, 22%, range 0.5–60.9 ng/mL), (OR 2.79, CI 95% 1.25–6.19, and *P* < 0.01). No statistically significant difference was found considering PFOS, PFOA, MEHP, and DEHP serum levels above the LOD between the two groups. A slightly, but not significant, difference between study group and control group was demonstrated for PFOS (32.4% in cases versus 18.1% in controls) and PFOA levels (43.2% in cases versus 18.1% in controls); noticeably, the upper range of PFOS levels in both cases and control groups was about one magnitude order higher than that of PFOA levels. The percentage of subjects with DEHP concentrations above the LOD was low in both groups (7.2% and 2.2% in cases and controls, resp.).

**Figure 1:**
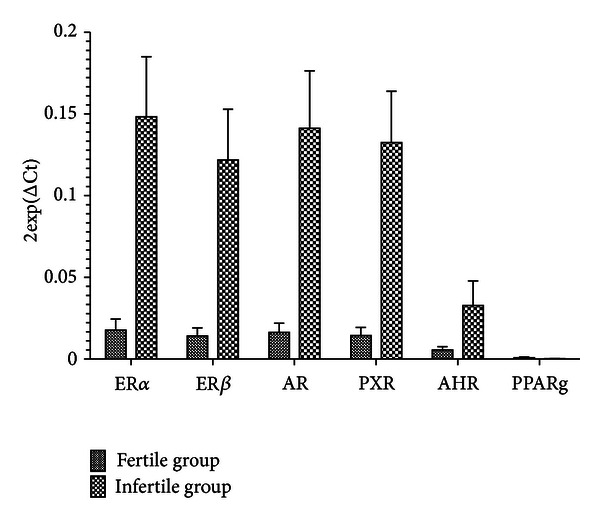
Nuclear receptors expression in infertile and fertile women.

Figure 1 and Table 2 show the cellular gene expression level of nuclear receptors in infertile and fertile women. All investigated NRs were expressed in PBMCs of both infertile and fertile women, with marked interindividual variability in both groups. Infertile patients showed gene expression levels of ER*α*, ER*β*, AR, and PXR significantly higher than the control group. The expression of AhR and PPAR*γ* did not show significant difference between the two groups.

Tables 3 and 4 point out the correlation between biomarkers of exposure and biomarkers of effects. A strong correlation was found between serum BPA concentration and ER*α* (*r* = 0.467; *P* < 0.0005), ER*β* (*r* = 0.474; *P* < 0.0005), and AR (*r* = 0.444; *P* < 0.0005) expression in infertile women. A positive correlation was also found in the control group (ER*α* *r* = 0.415, *P* < 0.05; ER*β* *r* = 0.371; *P* < 0.05; AR *r* = 0.378; *P* < 0.05). A positive association between BPA levels and AhR (*r* = 0.335; *P* < 0.005) and PXR expression (*r* = 0.429; *P* < 0.0005) was also demonstrated among infertile women, but not in controls. PFOS concentration positively correlated with AR (*r* = 0.236; *P* < 0.05) and PXR expression (*r* = 0.239; *P* < 0.05) in infertile women, while no association between PFOS exposure levels and NR expression was found in the control group. Only PFOA levels showed negative correlations with AhR (*r* = −2.242; *P* < 0.05) in infertile women. A positive correlation was found also between MEHP and ER*α* (*r* = 0.388; *P* < 0.005), ER*β* (*r* = 0.398, *P* < 0.005), AR (*r* = 0.366; *P* < 0.005), AhR (*r* = 0.291; *P* < 0.05), and PXR expression (*r* = 0.364; *P* < 0.005) in the infertile group, but not in the control group. No correlation was found between DEHP levels and the expression of all considered nuclear receptors.

## 4. Discussion

Our study underlines the relationship between environmental exposure and female infertility and the need to integrate internal exposure measurements with relevant indicators of biological and clinical effects [11]. Our results show how infertile women have higher levels of BPA when compared to healthy fertile women. The infertile group showed also significantly higher expression in PBMC of several NRs (ER*α*, ER*β*, AR, and PXR) that regulate endocrine pathways and are also potential EDC targets. BPA serum concentration showed a positive correlation with ER*α*, ER*β*, and AR in both fertile and infertile women, although the strength of this association appeared higher in the infertile group; moreover, in infertile women, BPA showed a specific, positive correlation with AhR and PXR expression. PFOS concentration showed a positive correlation with the expression of AR and PXR only in the infertile group. We investigated EDC that is still in use, widespread in living environment, and still receiving limited attention in environment and health surveillance.

BPA is a ubiquitous contaminant but is also considered a nonpersistent compound in our environment [12, 13]. Many studies on BPA focus on urinary excretion and in some cases make a distinction between BPA and its excreted metabolite BPA-glucuronide [13]. We decided to consider the serum level of total BPA as an indicator of BPA presence in the organism, as a result of repeated and prolonged uptake of the compound from the living environment. In 2010, the European Food Safety Authority has comprehensively reviewed the toxic effects of BPA, underlining how further data are necessary to clarify its association with human reproductive disorders [13]. BPA is considered as an agonist of estrogen receptors alpha (ER*α*) and beta (ER*β*) [14], but it could also interact with androgen receptors (ARs) [15]. Our data support the hypothesis that BPA may influence the expression of different NR involved in hormone response pathways and/or in steroid biosynthesis. This association is supported by recent experimental studies showing that BPA may alter ovarian steroidogenesis [16, 17], early events of uterine implantation, oocyte quality, and estradiol response to gonadotropin stimulation [18].

The increased PFOS exposure rate of infertile subjects did not reach statistical significance in our study; nevertheless, PFOS concentration showed a positive correlation with the expression of AR and PXR only in the infertile group. Both NRs are significantly upregulated in our infertile group, and a contribution of PFOS to such upregulation should not be ruled out. The percentage of subjects with PFOS concentration > LOD was found to be higher than the one reported by the study of Kannan et al. [19] and Ericson et al. [20] but comparable to those found by Yeung et al. [21], underlining the high variability of PFOS internal exposure, possibly related to factors influencing the persistent PFOS binding to plasma proteins [4, 19]. Toxicological and epidemiological evidence suggests a possible link between PFOS exposure and reproductive problems [22], including reduced oocyte fertilization capacity [9] and prolonged time to pregnancy [23].

Our study showed a widespread internal exposure of the women enrolled in the study to MEHP, as demonstrated by the fact that it is the only EDC consistently detected also in women of the control group. This result is consistent with previous biomonitoring studies [24]. However, a positive correlation between MEHP exposure levels and NR expression was found only in the infertile group. The emerging literature has demonstrated that exposure to DEHP and MEHP is associated with smaller preovulatory follicles, anovulation or delayed ovulation, longer estrous cycles, decreased synthesis of estradiol, decreased serum progesterone levels, and increased serum follicle-stimulating hormone (FSH) levels [25]. Beyond their well-described reprotoxic effects, phthalates are also suspected to interact with different members of the nuclear receptor (NR) superfamily or to act via their pathways by modulating expression of some nuclear receptors and their targets in various organ models [26].

The significantly increased expression of ER*α*, ER*β*, AR, and PXR in infertile women may be a direct consequence of endocrine-related reproductive disorders. Estrogens, whose functions are mediated by ER*α* and ER*β*, play a major role in steroidogenesis, follicular growth, ovulation, and endometrial cycle. AR is expressed in granulosa cells, oocytes, and in theca-insterstitial cells [27]. Its function is essential in optimizing follicular growth, final follicle development, and ovulation [28]. PXR indirectly mediates steroid hormone functions, playing an important role in their metabolism [29]. Therefore, a condition of altered ovarian function and/or endocrine signaling between the ovary and other reproductive tissues may be connected to NR dysregulation. Any change in NR expression may represent a relevant and plausible biomarker of effective systemic exposure to EDC [11].

Our study showed a strong positive correlation between BPA serum concentration and ER*α*, Er*β*, and AR expression both in infertile and fertile groups. Melzer et al. provided the first report regarding the relationship between BPA exposure and NR gene expression in humans, finding higher expression of ER*α* and ER*β* gene in PBMC of men associated with higher urinary BPA levels [30]. To date, our study is the first one investigating the expression of a comprehensive panel of NR in relation to internal exposure to BPA, as well as other EDCs, showing an NR upregulation. Infertile women showed a specific positive correlation between BPA and AhR and PXR expression. AhR is known as the “dioxin receptor,” but it may also play an important role in female reproduction. Studies on mice revealed that it is expressed in oocytes, granulose, and theca cells and that it is involved in ovarian follicle growth and estradiol biosynthesis [31]. Most importantly, AhR is involved in the cross-regulation of other NRs, in particular ERs [32]. PXR expression emerges as a potential biomarker related to EDC exposure and infertility, since it is enhanced in infertile women in association to either BPA and PFOS exposure. The upregulation of PXR is biologically plausible, since this “sensor” NR is involved in the metabolism of xenobiotics and endogenous compounds, including steroids [29]. It may be noteworthy that PFOA behaved differently than PFOS, as its internal levels are negatively correlated with PXR in fertile women and with AhR in infertile patients. Indeed, Kraugerud et al. [33] suggest that the toxicological patterns of PFOS and PFOA might not overlap.

Finally, the expression of PPAR*γ* in PBMC was low and did not show any meaningful change, indicating that it is not a reliable biomarker for EDC in women at childbearing age.

We are aware of the potential limitations of our study related to the presence of potential confounding factors, the sample size, and the cross-sectional design. We checked the presence of potential confounding factors, such as age, smoking, BMI, metabolic disease conditions, or special dietary habits (celiac disease, vegetarianism). No significant differences between infertile and control fertile women were found. Our sample size (both infertile and control groups) has limited the statistical power of the study in detecting small differences, assuming that EDC could have multiple targets and can affect woman reproduction in more than one way [2, 34].

To date, our data cannot completely state the effective role of EDC in the onset of reproductive disorders but point out how specific NRs could be relevant markers to be used in EDC risk assessment. Even if the currently available studies are still insufficient to support a full risk characterization of EDC, evidence prompts to further investigation to address knowledge gaps and precautionary actions against excess exposure to specific compounds. It is really important to consider human exposure to multiple compounds as a real-life scenario to provide special attention to substances that are still widely present in consumer products and to integrate exposure measurements with relevant indicators of biological effects. Such evidence should be seen as sufficient grounds to take precautionary action to substitute potential endocrine disruptors as well as endocrine disruptors.

## Figures and Tables

**Table 1:** Proportion of samples with EDC levels > LOD in both infertile and fertile groups.

Endocrine disruptor	Infertile group (*n* = 111)	Fertile group (*n* = 44)	*P* value	OR (CI 95%)
BPA	50 (45)	10 (22.7)	0.01	2.79 (1.25–6.19)
PFOS	36 (32.4)	8 (18.2)	0.11	2.16 (0.91–5.12)
PFOA	48 (43.2)	16 (36.4)	0.47	1.33 (0.64–2.65)
MEHP	73 (65.8)	27 (61.4)	0.71	1.21 (0.59–2.49)
DEHP	8 (7.2)	1 (2.3)	0.45	1.26 (0.40–27.5)

**Table 2:** Cellular gene expression level of nuclear receptors in infertile and fertile women.

NR	Infertile group (*n* = 111)	Fertile group (*n* = 44)	*P* value
ER*α*	0.15 ± 0.31	0.02 ± 0.04	0.02
ER*β*	0.12 ± 0.27	0.01 ± 0.03	0.02
AR	0.14 ± 0.30	0.02 ± 0.03	0.02
PXR	0.13 ± 0.27	0.014 ± 0.03	0.01
AhR	0.03 ± 0.13	0.005 ± 0.01	0.23
PPAR*γ*	0.0002 ± 0.0004	0.0007 ± 0.0003	0.15

**Table 3:** Correlation between ECD and receptor expression in infertile group.

	ER*α*	ER*β*	AR	PXR	AHR	PPAR*γ*
BPA	0.467*	0.474*	0.444*	0.429*	0.335*	0.091
PFOS	0.214	0.209	0.236*	0.239*	0.090	0.122
PFOA	−0.203	−0.203	−0.203	−0.191	−0.242*	−0.150
MEHP	0.388*	0.398*	0.366*	0.364*	0.291*	0.082
DEHP	−0.101	−0.179	−0.134	−0.145	−0.157	−0.151

*P < 0.05.

**Table 4:** Correlation between ECD and receptor expression in fertile group.

	ER*α*	ER*β*	AR	PXR	AHR	PPAR*γ*
BPA	0.415*	0.371*	0.378*	0.331	0.300	−0.155
PFOS	−0.086	0.007	−0.031	−0.209	−0.086	−0.059
PFOA	−0.259	−0.185	−0.176	−0.364*	−0.310	0.089
MEHP	0.342	0.157	0.170	0.230	0.239	0.007
DEHP	0.240	0.240	0.240	0.222	0.257	0.186

*P < 0.05.
